# *Chondrites* isp. Indicating Late Paleozoic Atmospheric Anoxia in Eastern Peninsular India

**DOI:** 10.1155/2014/434672

**Published:** 2014-01-30

**Authors:** Biplab Bhattacharya, Sudipto Banerjee

**Affiliations:** ^1^Department of Earth Sciences, Indian Institute of Technology Roorkee, Roorkee, Uttarakhand 247667, India; ^2^Department of Geology, Hooghly Mohsin College, Chinsurah, Hooghly, West Bengal 712101, India

## Abstract

Rhythmic sandstone-mudstone-coal succession of the Barakar Formation (early Permian) manifests a transition from lower braided-fluvial to upper tide-wave influenced, estuarine setting. Monospecific assemblage of marine trace fossil *Chondrites* isp. in contemporaneous claystone beds in the upper Barakar succession from two Gondwana basins (namely, the Raniganj Basin and the Talchir Basin) in eastern peninsular India signifies predominant marine incursion during end early Permian. Monospecific *Chondrites* ichnoassemblage in different sedimentary horizons in geographically wide apart (~400 km) areas demarcates multiple short-spanned phases of anoxia in eastern India. Such anoxia is interpreted as intermittent falls in oxygen level in an overall decreasing atmospheric oxygenation within the late Paleozoic global oxygen-carbon dioxide fluctuations.

## 1. Introduction

Trace fossils provide signatures of animal responses to various ecological controls, including salinity and its fluctuations, sedimentation rate, oxygenation, availability of food, and temperature. Oxygen deficiency is common in deep, quiet water environments, where fine-grained sediments accumulate slowly. In shallower marginal marine conditions, atmospheric oxygen content regionally controls the availability of oxygen near the sediment-water interface. Besides, decomposition of large quantities of terrigenous organic detritus in wave-dominated estuaries may cause localized lowering of oxygen levels near the sediment-water interface [1]. Low oxygen concentration leads to substantial reduction in the size of the trace fossils and their diversity [2], accompanied by dominance of deposit-feeding burrows that maintain an open conduit to the sediment-water interface [3]. Trace fossil assemblage found in such settings includes *Chondrites*, *Zoophycos*, *Teichichnus*, and *Trichichnus* [2–4]. Monospecific assemblages of *Chondrites *suggest poorly oxygenated bottom waters [5] and are considered to be associated with prolific marine conditions of deposition [2, 3]. The tracemaker of *Chondrites *may be able to live in the aerobic/anoxic interface as a chemosymbiotic organism that pumps methane and hydrogen sulphide from the sediments [6].

The present paper reports the occurrence of *Chondrites *isp. from the sedimentary successions of the early Permian Barakar Formation (in lower Gondwana Supergroup), exposed along the Kudaposi Nala section in the Talchir Basin and the Khudia Nala section in the Raniganj Basin, eastern peninsular India. Geographic distance between these two areas is ~400 km. This paper describes the occurrence of different ichnospecies of *Chondrites *from two widely apart Gondwana basins and correlates that with the fluctuations of atmospheric oxygen level during the late Paleozoic period.

## 2. Geological Background

**Figure 1:**
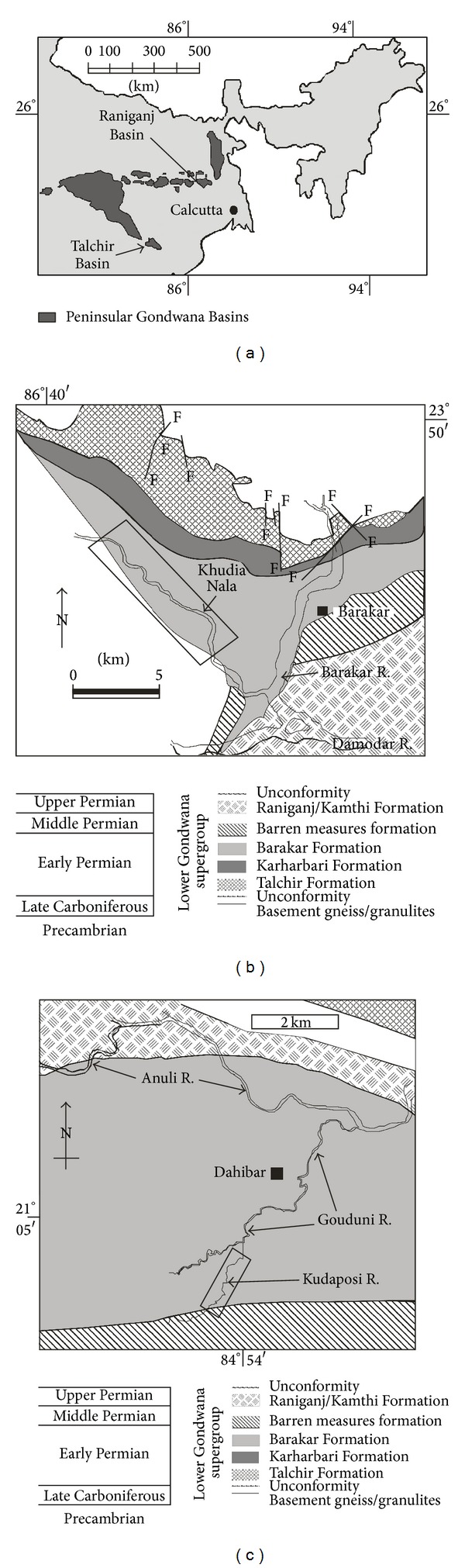
(a) Map of eastern India showing the distribution of the Gondwana Basins. Note the location of the Raniganj Basin and the Talchir Basin. (b) Geological map of the north-western part of the Raniganj Basin, showing the distribution of different lithounits. (c) Detailed geological map of the central part of the Talchir Basin, showing the distribution of different lithounits. The legend shows the ages of the lithounits. Study areas are shown by rectangles in both the maps ((b) and (c)).

**Figure 2:**
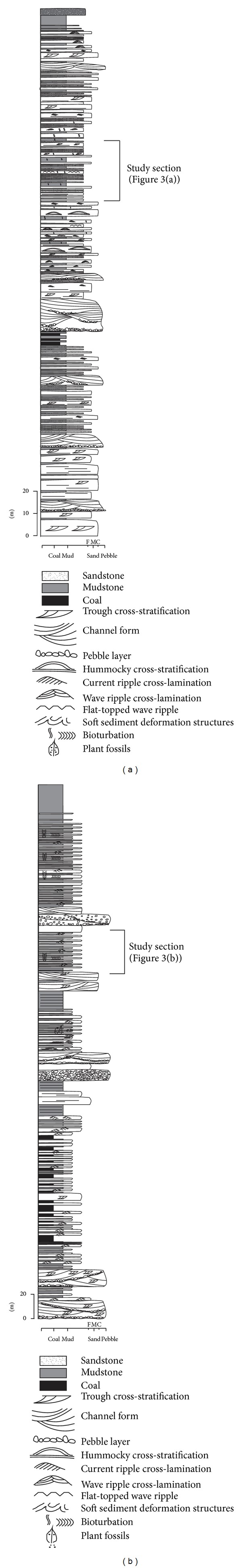
Generalized sedimentary logs of the Barakar Formation in (a) Raniganj Basin and (b) Talchir Basin. The study sections are marked and are detailed in Figure 3(a) (Khudia Nala) nad Figure 3(b) (Kudaposi Nala), respectively.

Barakar Formation (early Permian) within the Gondwana sedimentary succession is the main coal-producing horizon in Indian Subcontinent. Earlier studies recorded a braided-meandering fluvial depositional system with peat-forming mires in the low-lying marshy floodplains that produced thick succession of sandstone-shale-coal cyclothems within the Barakar Formation [7]. Recent sedimentological analyses from multiple Gondwana basisn of India suggested strong influence of marine wave and tide on fluvial sedimentation during the deposition of middle-upper Barakar succession [8–10], indicating a fluvio-marine interactive deltaic/estuarine depositional setting. The Barakar Formation in the Raniganj Basin in the Koel-Damodar Valley and the Talchir Basin in the Mahanadi Valley (Figure 1), comprises of thick sandstone-shale-coal deposits (Figure 2) manifesting similar depositional history. The lower parts of the sedimentary successions in both the basins bear thick coal seams and represent braided-fluvial depositional setting (Figure 2).

**Figure 3:**
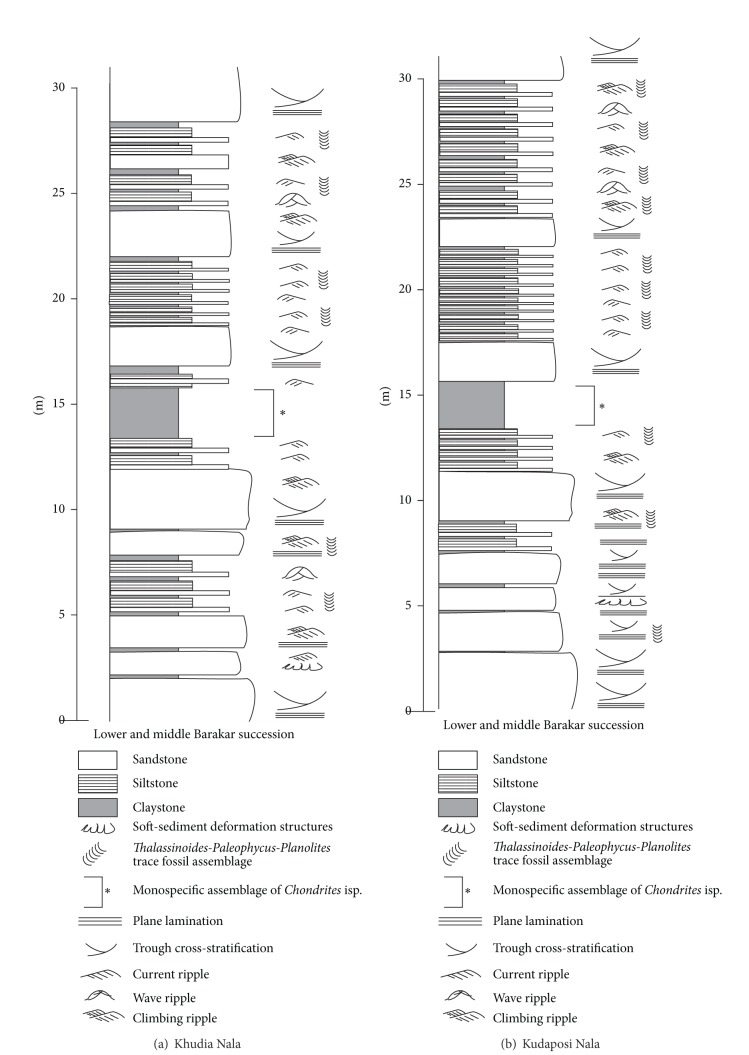
Vertical sedimentary logs of the upper Barakar succession in (a) Khudia Nala, Raniganj Basin, and (b) Kudaposi Nala, Talchir Basin. Note the occurrence of the claystone beds containing monospecific assemblage of *Chondrites* isp.

**Figure 4:**
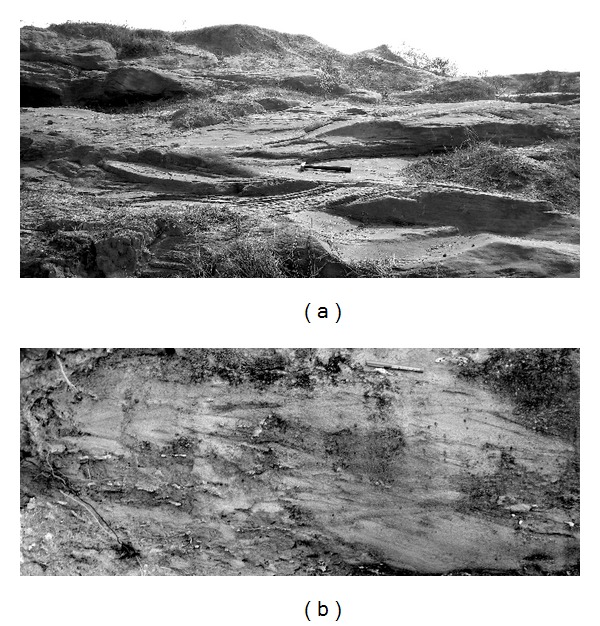
(a) Trough cross-stratified sandstone facies within Barakar Formation, Khudia Nala, Ranjiganj Basin. Length of the hammer is 30.5 cm. (b) Amalgamated sandstone beds showing multiple sets of planar and trough cross-stratification, Barakar Formation, Kudaposi Nala, Talchir Basin. Length of the pen is 14.6 cm.

**Figure 5:**
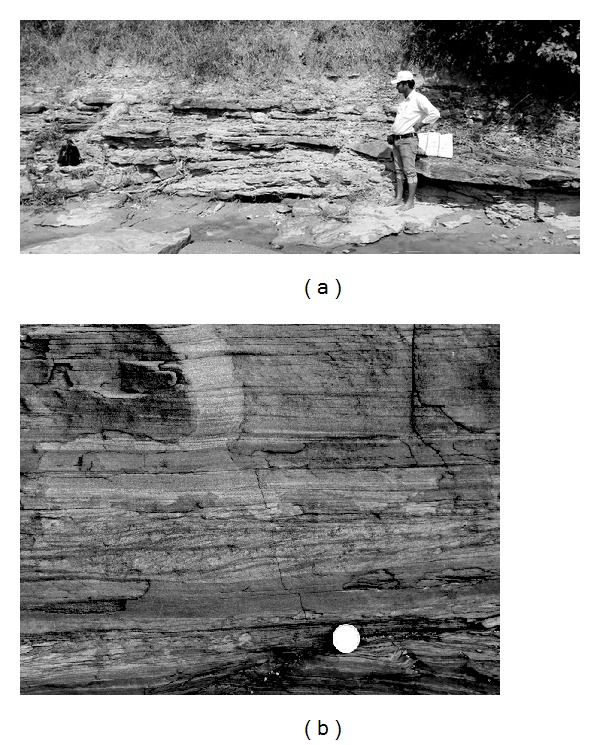
(a) The sandstone-siltstone-claystone facies exposed in the Kudaposi Nala, Talchir Basin. (b) Details of the sandstone-siltstone-claystone facies, exposed in the Khudia Nala, Raniganj Basin. Diameter of the coin is 2.3 cm.

The studied rocks preserved in both the Kudaposi Nala section, Talchir Basin, and the Khudia Nala section, Raniganj Basin (Figure 1), represent part of the upper Barakar sedimentary succession (Figures 2 and 3). The upper Barakar sedimentary successions of both the Damodar Valley and the Mahanadi Valley have been assigned the age of Kungurian (post-Artinskian) with an approximate time frame of 278–271 Ma [11]. The studied rocks are characterized by thick-bedded (1–2.5 m thickness), coarse-grained, trough cross-stratified to plane-laminated sandstone facies (Figures 4(a) and 4(b)), overlain by cm thin sandstone-siltstone-claystone alternations facies (Figures 5(a) and 5(b)). Thickness of the sandstone-siltstone-claystone alternation facies varies from 1.5 m to 4.3 m. The sandstone beds within this facies are characterized by wave ripples and wave-modified ripples, tidal bundles represented by mud-draped foresets, climbing ripples, flaser beddings, bidirectional ripples, mutually opposite cross-strata sets, vertical and lateral accretion of cross-strata sets with prominent reactivation surfaces, and so forth. Each sandstone bed grades upward to a siltstone/claystone. The claystone beds are occasionally very thick (thickness 1–1.8 m) and are characterized by abundant *Chondrites *isp. on the bedding plane. The sandstone/siltstone-claystone heteroliths that underlie and overlie such thick claystone beds are often characterized by sparse to abundant trace fossil associations, dominantly *Planolites *isp., *Thalassinoides *isp., and *Paleophycus *isp., along with abundant plant fossils. The successions show coarsening upward trend with gradual increase in the deposition of sandstone towards top, and finally overlain by a thick-bedded, trough cross-stratified, coarse-grained sandstone facies.

The sedimentary facies architecture closely resembles those described from Barakar sedimentary succession of the Satpura Basin and the Raniganj Basin, respectively [9, 10]. The sedimentation patterns changed from a basal braided channel to an upper meandering channel system, where rapid migration of the channels and lateral accretion/coalescing of bars led to deposition of thick trough cross-stratified to plane laminated sandstone [8]. Open marine tide and wave influences produced multiple fining upward successions with characteristic sedimentary features and indicate episodic drowning of the fluvial channels by marine water [10]. Dominant fining-upward succession indicates overstepping overbank levees within a meandering fluvial-marine interactive estuarine setting [12, 13]. A coarsening-upward succession comprising of coarse-grained channel-filling sandstone facies overlies this succession, indicating progradation of the meandering channel system over the estuarine deposits.

## 3. Ichnogenus* Chondrites *Sternberg

*Chondrites *Sternberg is considered as an infaunal deposit-feeding system [14]. It comprises a regularly branching tunnel system consisting of a small number of master shafts open to the surface, which ramify at depth to form a dendritic network [14–17]. In the present paper, we describe four different species of *Chondrites *from the Barakar Formation, namely, *Chondrites patulus *(from Kudaposi Nala), *Chondrites targionni *(from both Kudaposi Nala and Khudia Nala), *Chondrites affinis *(from both Kudaposi Nala and Khudia Nala), and *Chondrites recurvus *(from Khudia Nala). In both the study sections, all the ichnoforms are hosted in fine-grained, massive claystone, mostly preserved as epichnial, sand/silt-filled ridges on the claystone bedding surfaces. The following is a description of all the ichnospecies recorded from the two areas under study.

### 3.1. Ichnospecies *Chondrites patulus* (Figures 6(a) and 6(b))

These are small, straight, unlined, branching burrow system, preserved on the claystone bedding surfaces. These ichnoforms show filling by fine-grained sandstone/siltstone and characteristically lack spreiten laminae. Offshoots branch out from the main stem on both sides making obtuse angle with the stem. Individual branches are 0.25 to 0.3 cm wide and 1.2 cm in length. Offshoots do not show any further branching.

### 3.2. Ichnospecies *Chondrites targionii* (Figures 6(b) and 6(c))

This ichnospecies is represented by straight to dendritic, unlined, smooth walled burrow system with few branches, preserved on the claystone bedding surface. Traces are parallel to bedding surface and radiate at an angle varying from low to high angle. Secondary branching is absent. Length of the branches varies between 1.2 and 1.5 cm and width up to 0.22 cm. Burrow fills are commonly coarser-grained sediments with respect to the host sediments. Spreiten lamellae are characteristically absent.

### 3.3. Ichnospecies *Chondrites affinis* (Figures 6(a), 6(b), and 6(c))

These are represented by relatively larger, epirelief, bedding parallel concave tubes. These are preserved within claystone as straight to curved sand/siltstone ridges with locally preserved spreiten lamellae. The tubes are unlined and rarely show branching. Main stem is 4.1–6.5 cm in length and 0.25 cm wide with the branches varying in length between 0.4–0.8 cm and 0.25 cm in width. Secondary branching is absent. The tubes locally overly other such tubes.

### 3.4. Ichnospecies *Chondrites recurvus* (Figures 6(a) and 6(c))

These ichnoforms are represented by short, curved, epirelief traces. Branching intensity is relatively high with respect to other ichnoforms in the study area. The branches are peculiarly curved following the main stem. Length of the main stem is approximately 2.5 cm whereas the branches are up to 0.4 cm in length. Locally these traces are not clear due to poor preservation of the off-suits.

## 4. Discussion

The ichnoform  *Chondrites *is considered as an indicator of poor oxygenation (dysaerobic-exaerobic) conditions [5, 18–22] and has been described from several late Paleozoic sedimentary successions, including shallow marine argillites, storm-led sediments, and flysch deposits. These three-dimensional, branched, straight to curved, dendritic burrow systems are considered as deposit-feeding traces [14]. These were reinterpreted as complex, agrichnial traces based on their chemosymbiotic activities within predominantly anoxic environment [5]. Polychaete worms are considered as the most likely producers of *Chondrites*, as they can tolerate oxygen-deficient conditions [23–26]. *Chondrites*-like deposit-feeding burrows with open connections to the sediment-water interface are typical of facies associated with extremely low oxygen levels in interstitial and bottom waters [3].

Estuaries are characterized by numerous ecological stresses on potential trace-making organisms, including fluctuating salinity levels, high water turbidity, rapid sedimentation rates, and low oxygen levels in bottom and interstitial waters. These paleoecological factors control the trace fossil assemblage in estuaries. Trophic generalists are able to adapt their feeding strategy in response to changing environmental controls under brackish-water settings, producing complex burrow morphologies characteristic of marine ecological system [27]. This is the case observed in the estuarine deposits of the late Paleozoic upper Barakar Formation, eastern India. Thick claystone beds within the sandstone-siltstone-claystone alternation facies represent deposition in low-lying, swampy, interdistributary plains. This claystone bears the monospecific assemblage of *Chondrites *ichnoforms. The overlying and underlying sequence of thin-bedded sandstone-mudstone heteroliths, formed by open marine tide-wave reworkings near the distributary mouth, containing sparse to dense assemblage of *Planolites *isp., *Thalassinoides *isp., and *Paleophycus *isp. The overall *Thalassinoides-Planolites-Chondrites *ichnocoenoses is characteristic of estuarine setting.

The *Chondrites*-bearing claystone beds attest to dominant marine inundations (maximum flooding surface (MFS)) of the estuary, with prevalence of anoxic phases in the ambience. Occurrence of abundant *Chondrites *ichnoassemblage in multiple narrow but identical sedimentary horizon (claystone) in widely apart geographic locales in eastern peninsular India points to multiple short but predominant anoxic phases in the depositional realm that possibly persisted all over the eastern peninsular India during the end early Permian time. As the overlying and underlying sediments contain plenty of plant fossils and this part of the lithosuccession is devoid of any intermittent coal layers; hence, the possibility of development of low oxygenation due to decomposition of large organic detritus is negated. So, such localized anoxia within the upper Barakar estuarine system can be correlated with fluctuations in the atmospheric oxygen level. Sequential transition from monospecific *Chondrites *assemblage to a mixed *Planolites*-*Thalassinoides* assemblage indicates gradually increasing oxygenation in the ambience [4, 5].

**Figure 7:**
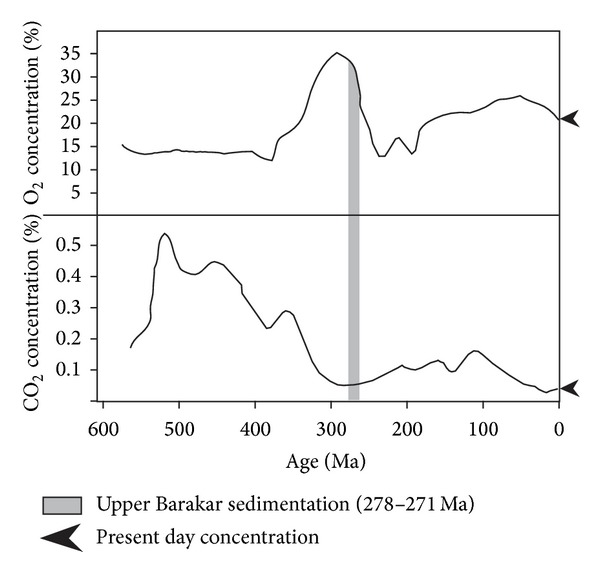
Plot showing concentration of O_2_ and CO_2_ in the atmosphere during the Phanerozoic time (modified after 31, 32). Note the O_2_ and CO_2_ concentrations during the upper Barakar sedimentation (278–271 Ma).

Atmospheric oxygen levels show wide fluctuations during the late Paleozoic time. The oxygen level reached a maximum of 35% at the end Carboniferous causing enhancement of respiration and gigantism in organisms. This was followed by a significant drop to 15% at the end Permian leading to mass extinction at the Permo-Triassic boundary. Concomitantly, the carbon dioxide content was raised through the Devonian and Carboniferous, followed by an approximately tenfold reduction during the middle to late Paleozoic time [28, 29]. During Permian, extensive coal deposition resulted from lesser extent of decomposition of terrestrial plant ecosystems and increased carbon fixation by carbon-reducing organisms. Such disequilibrium between free and stored carbon concentration, extensive oxygen release due to post-Silurian terrestrialization by plants together with changes in continental weathering, organic carbon deposition, and biotic decomposition dramatically modified the atmospheric carbon dioxide and oxygen levels [28, 30]. The time frame 278–271 Ma (upper Barakar sedimentation) demarcates a steady fall in the atmospheric oxygen level (~32–25%) accompanied by a very slow-rising carbon dioxide concentration (<0.1%) in global scale (Figure 7) [31, 32]. Record of anoxia demarcated by the *Chondrites* ichnoassemblage, followed by *Planolites*-*Thalassinoides *ichnoassemblages in the overlying and the underlying successions, within the upper Barakar succession in eastern India can be correlated with intermittent drops in oxygen level within a steady phase of decreasing oxygenation within an overall fluctuating atmospheric condition during the early Permian period.

## 5. Conclusion

Postglacial marine invasion of different terrestrial areas within the Gondwanaland during the Permian is well known, and the Permian Gondwana sediments from India are not exception to this. Under this framework, in recent times, coal-bearing terrestrial-fluvial sequences of the Barakar Formation are reinterpreted as marine wave-tide influenced deltaic/estuarine deposits, based on different sedimentological and ichnological attributes. Different ichnoforms of the *Chondrites *isp. are reported for the first time from the upper Barakar succession, which provides further support favouring such marine inundation. Abundance of this typical marine ichnocoenoses in laterally distant areas within several narrow time frames demarcate short phases of anoxia in eastern India during end early Permian period. In this paper, we correlate such anoxic fluctuations with periods of falling-oxygenation within the global oxygen-carbon dioxide fluctuation in the late Paleozoic atmosphere. Record of such atmospheric fluctuation and resultant sediment-organism interaction pattern will be useful in the understanding and the reconstruction of the paleoecological and paleoenvironmental changes within the Permian Gondwanaland-Tethys system.

## Figures and Tables

**Figure 6:**
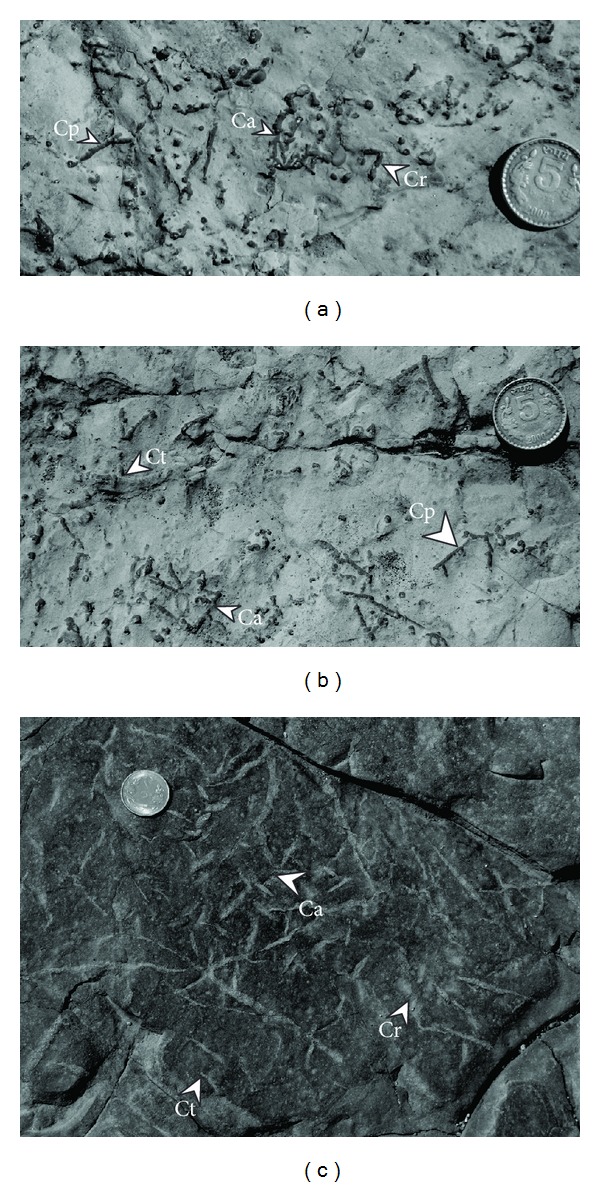
Claystone bedding surfaces within upper Barakar succession, showing associations of *Chondrites patulus* (Cp), *Chondrites targionii* (Ct), *Chondrites affinis* (Ca), and *Chondrites recurvus* (Cr). (a) and (b) are from Kudaposi Nala, Talchir Basin, and (c) is from Khudia Nala, Raniganj Basin. Diameter of the coin is 2.3 cm in (a) and (b) and 2.5 cm in (c).
